# Pro‐inflammatory signals induce 20α‐HSD expression in myometrial cells: A key mechanism for local progesterone withdrawal

**DOI:** 10.1111/jcmm.16681

**Published:** 2021-06-10

**Authors:** Lubna Nadeem, Rathesh Balendran, Anna Dorogin, Sam Mesiano, Oksana Shynlova, Stephen J. Lye

**Affiliations:** ^1^ Lunenfeld Tanenbaum Research Institute Mount Sinai Hospital Toronto ON Canada; ^2^ Department of Reproductive Biology Case Western Reserve University Cleveland OH USA; ^3^ Department of Physiology University of Toronto Toronto ON Canada; ^4^ Department of Obstetrics & Gynecology University of Toronto Toronto ON Canada

**Keywords:** 20α‐HSD, AP‐1, labour, myometrium, NF‐кB, preterm birth, progesterone metabolism

## Abstract

Metabolism of progesterone (P4) by the enzyme 20α hydroxysteroid dehydrogenase (20α‐HSD) in myometrial cells is postulated to be a mechanism for P4 withdrawal, which occurs concomitant to uterine inflammation (physiologic or infection‐induced) and associated activation of transcription factors: NF‐кB and AP‐1, common to term and preterm labour. We found that 20α‐HSD protein is significantly increased in human myometrium during term labour, and in mouse uterus during term and preterm labour. Treatment of human myometrial cells with the pro‐inflammatory mediators, lipopolysaccharide (LPS, mimicking infection) and 12‐O‐tetradecanoylphorbol‐13‐acetate (TPA, mimicking inflammation), induced 20α‐HSD gene expression and increased 20α‐HSD protein abundance. LPS treatment decreased P4 release into the culture medium and resulted in up‐regulation of GJA1 in the hTERT‐HM cells. The NF‐кB /AP‐1 transcription factors mediated effects of LPS and TPA on 20α‐HSD gene transcription. Both pro‐inflammatory stimuli induced 20α‐HSD promoter activity in LPS/TPA‐treated cells which was significantly attenuated by inhibition of NF‐кB (JSH: 20 µM) or AP‐1 signalling (T5224: 10 µM). Deletion of NF‐кB consensus sites abrogated LPS‐mediated promoter induction, while removal of AP‐1 sites reversed the TPA‐mediated induction of 20α‐HSD promoter. We conclude that inflammatory stimuli (physiologic or pathologic) that activate NF‐кB or AP‐1 induce 20α‐HSD transcription and subsequent local P4 withdrawal resulting in up‐regulation of GJA1 and activation of myometrium that precedes labour.

## 1 INTRODUCTION

The steroid hormone progesterone (P4) is essential for the establishment and maintenance of pregnancy, and in all eutherian species examined so far, P4 withdrawal by decline in P4 levels or disruption of P4 signalling initiates labour. Human parturition that occurs without a systemic decline in maternal P4 levels is speculated to be triggered by local P4 withdrawal in the uterine tissues.^1^, ^2^ Local P4 withdrawal can occur by changes in signalling mediated by the nuclear P4 receptor (PR) isoforms, PR‐A and PR‐B or by increased metabolism of P4 to a PR‐inactive form in uterine target cells. Our previous data suggest a combination of these mechanisms whereby local metabolism/inactivation of P4 within the myometrium leads to the loss of P4/PR‐B function and dominance of ligand‐independent PR‐A activity that induces expression of genes encoding contraction‐associated proteins (CAPs) that augment myometrial contractility.^2^ Local intracrine P4 withdrawal can be mediated by the P4‐metabolizing enzyme, 20α‐hydroxysteroid dehydrogenase (20α‐HSD),^1^, ^2^ which belongs to the aldo‐keto reductase (AKR) superfamily and acts on a variety of substrates, including steroid hormones and endogenous prostaglandins.^3^, ^4^ In particular, human 20α‐HSD (encoded by *AKR1C1,* murine homologue*: Akr1c18*) catalyses the conversion of P4 into 20α‐hydroxyprogesterone (20α‐OHP), a relatively weak progestin compared with P4.^5^ It has been reported earlier that compared to early gestation (13th week), the P4/20α‐OHP ratio is decreased in term (40‐42nd week) human myometrium suggesting increased P4 metabolism with advancing gestation.^5^

The association of 20α‐HSD with labour is evident from studies in rodents. In rats, expression of 20α‐HSD in ovaries/corpus luteum negatively correlated with the circulating P4 levels, with luteolysis being associated with increased 20α‐HSD expression and the fall in P4 that initiates labour.^6^, ^7^ In mice, the knockout of the transcription factor (TF) STAT5b caused early abortion due to increased expression and activity of 20α‐HSD in corpus luteum and subsequent P4 withdrawal.^8^, ^9^ Moreover, 20α‐HSD gene knockout in mice increased P4 levels and delayed parturition,^9^, ^10^ suggesting a central role of the enzyme in labour onset. This may be critical in facilitating local P4 withdrawal in the myometrium to trigger labour. In lower mammals despite the decline in peripheral P4, maternal P4 levels at term remain well above the dissociation constant (Kd) for binding to PRs.^11^ Similarly in humans, increased expression and abundance of myometrial 20α‐HSD^1^ has been associated with local withdrawal of P4 and P4/PR function in myometrium.^1^, ^2^ It is plausible that PTB is caused by premature induction of 20α‐HSD activity in myometrial cells, thus understanding how 20α‐HSD is regulated is of paramount importance.

The mechanisms by which 20α‐HSD is up‐regulated in term myometrium^1^, ^2^ in the presence of high P4 levels is unclear since its transcription is suppressed by P4/PR signalling during gestation.^12^, ^13^ A possible explanation is the presence of other factors that induce 20α‐HSD expression in the term myometrium. Physiologic inflammation^14^ as a result of maternal and foetal causes, such as mechanical stretch^15^ and foetal lung maturation,^16^, ^17^, ^18^ that increase cytokines/chemokine levels and corresponding immune cells infiltration might be one such factor.^19^, ^20^ There is ample evidence that in both mice and humans, pro‐inflammatory transcription factors (TFs), AP‐1^21^, ^22^ and NF‐кB,^16^ are induced in the myometrium during term and preterm labour. In humans, cervical expression of AP‐1 and NF‐кB has shown to be increased prior to parturition.^23^ Since systemic P4 levels remain elevated in humans prior to and during labour, which negatively regulates 20α‐HSD expression [reviewed in^24^], we hypothesized that uterine inflammation causes 20α‐HSD induction in myometrium and that the pro‐inflammatory TFs, AP‐1 and/or NF‐кB, play a role in the transcriptional regulation of the gene encoding 20α‐HSD.

In this study, we examined the myometrial expression of 20α‐HSD in pregnant women and mice, in association with term and preterm labour and determined whether it is affected by pro‐inflammatory stimuli and by the activity of NF‐кB or AP‐1 TFs. We found that myometrial levels of 20α‐HSD increase in association with term and preterm labour and that the activation of NF‐кB or AP‐1 induces *AKR1C1* transcription. Our findings suggest a functional link between uterine inflammation and local P4 withdrawal that triggers labour.

## 2 MATERIALS AND METHODS

### 2.1 Ethical approval for human and murine studies

The study involving human tissues was approved by the Research Ethics Board of Mount Sinai Hospital, Toronto, Canada (REB #18‐0168‐A). All patients provided a written consent to participate in the study. All murine experiments were approved by the institutional Animal Care Committee (AUP # 21‐0164H). Hsd:ICR (CD‐1) outbred timed pregnant dams were purchased from Harlan Laboratories (http://www.harlan.com/) and housed under specific pathogen‐free conditions at the Toronto Centre for Phenogenomics (TCP) on a 12L:12D cycle and were administered food and water ad libitum.

### 2.2 Murine models of labour; Term labour (TL) model

Female mice were mated overnight with males, and the day of vaginal plug detection was designated gestational day (GD) 0.5 of pregnancy. The average time of delivery was the early morning of GD19. Mice were killed by carbon dioxide inhalation, and uteri were collected at 10 AM on all days with the exceptions of the labour sample (TL) that was collected once the animals had delivered at least one pup from average number of 14 in two uterine horns. Myometrial tissue was collected from 4 animals on GD8 (early gestation), GD15 (mid‐gestation), GD19 (term not in labour, TNIL), and GD19/20 (term labour, TL).

### 2.3 Preterm labour (PTL) models

#### 2.3.1 LPS‐induced PTL

The lipopolysaccharide (LPS) used for this study was isolated from Escherichia coli, serotype O55:B5 (Sigma‐Aldrich). On GD15, mice underwent mini‐laparotomy under general anaesthesia (isoflurane) with intrauterine infusion of 125 μg LPS in 100 μL of sterile saline (LPS group) or 100 μL sterile saline (Sham group) between two amniotic sacs close to the cervix. Animals (n = 3 per group) were killed during LPS‐induced PTL (12‐24 hours after the infusion) or 24 hours after a saline injection.

#### 2.3.2 RU486‐induced PTL

On GD15, two groups of mice were subcutaneously injected with either mifepristone (RU486, 17ß‐hydroxy‐11β‐[4‐dimethylaminophenyl]‐17‐[1‐propynyl]‐estra‐4,10‐dien‐3‐ne; Biomol International, 150 μg in 100 μL corn oil containing 10% EtOH, RU486 group) or vehicle (100 μL corn oil containing 10% EtOH, vehicle group) (n = 4 per group). PTL occurred in 24 ± 2 hours post‐RU486 treatment. Myometrial samples were collected from RU486 group during PTL after delivery of at least one pup, and 24 hours after vehicle injection from the control group.

### 2.4 Myometrial tissue collection

#### 2.4.1 Human tissues

Healthy pregnant women with a singleton pregnancy undergoing elective caesarean delivery at term (gestational age ≥37 weeks) were recruited as ‘term not in labour’ (TNIL, n = 5). Caesarean delivery of ‘term in labour’ (TL, n = 5) women was performed after the onset of labour (with regular uterine contractions at 10‐minutes interval and cervical dilatation >3 cm) for indications of foetal distress. Myometrial biopsy samples of approximately 1 cm^3^ were excised from the upper margin of the lower uterine segment post‐delivery and washed in ice‐cold PBS. For protein analysis, a small part of the biopsy was snap‐frozen in liquid nitrogen and stored at −80℃; the rest was immediately transferred to 10% neutral‐buffered formalin (NBF, Harleco) or 4% paraformaldehyde (PFA, Electron Microscopy Sciences), for 24 hours at 4℃, washed in PBS, dehydrated in increasing grades of ethanol and embedded in paraffin wax.

#### 2.4.2 Murine tissues

Mice were killed by carbon dioxide inhalation at specific gestational days and during TL or PTL. The part of uterine horn close to cervix from the horn from which a foetus was expelled (post‐partum tissue) was removed; the remainder was collected for analysis. For biochemical analyses, uterine horns were bisected longitudinally in ice‐cold PBS. The decidua basalis and decidua parietalis was removed as described previously.^25^ Myometrial samples were snap‐frozen in liquid nitrogen and stored at −80℃ for protein analysis. For immunohistochemistry analyses (IHC), one whole uterine horn was fixed in 10% NBF or 4% PFA and processed as described in (A) above.

### 2.5 Cell lines and cell culture

The human telomerase immortalized myometrial cells (hTERT‐HM) obtained from Dr Jennifer Condon^26^ were maintained in DMEM/F12, and human embryonic kidney cell line HEK293T, purchased from ATCC (cat # CRL‐3216™), was maintained in DMEM. Media were supplemented with 10% FBS, 100 IU/mL penicillin and 100 µg/mL streptomycin. All the reagents for cell culture were purchased from Invitrogen Canada Inc.

#### 2.5.1 In vitro treatments

LPS purified from *E coli* (serotype O55:B5, Cat# L2880), 12‐O‐tetradecanoylphorbol‐13‐acetate (TPA, Cat # P1585) and Progesterone (P4, Cat # P8783) were purchased from Sigma‐Aldrich. The NF‐кB inhibitor: JSH (cat #ab144824) was obtained from Abcam, and AP‐1 inhibitor: T5224 (Cat #22904) from Cayman Chemicals. LPS was reconstituted with ultrapure water, P4 with 70% ethanol, while TPA, JSH and T5224 were reconstituted with dimethyl sulphoxide (DMSO) as per manufacturer's instructions.

#### 2.5.2 Assessment of AKR1C1 mRNA and protein

The hTERT‐HM cells were serum‐starved in FBS‐free medium supplemented with 1% insulin selenium transferase (ITS), for overnight and then treated with 1) control (C, water) or LPS (1 µg/mL), 2) vehicle (V, 0.1% DMSO) or TPA (20 ng/mL), 3) serum‐free medium (SF) or LPS‐conditioned medium (CM) for 4 hours for RNA extraction or 24 hours for protein collection. The CM was prepared by treating the cells with LPS for 4 hours, and then, the LPS medium was removed, cells were washed with PBS^−/−^ (without calcium and magnesium addition) and then incubated in serum‐free medium (supplemented with 1% ITS) for 16 hours (overnight). The culture medium from cells was collected and named ‘CM’, enriched with secreted factors (cytokines and chemokines) from the LPS‐treated cells.

#### 2.5.3 Activation and Inhibition of NF‐кB

hTERT‐HM cells were serum‐starved as above and then treated with LPS (1 µg/mL) or control. Cell lysates for protein extraction were collected at 0, 15, 30 and 60 minutes to examine phosphorylation of IKBα. To validate the activity of NF‐кB inhibitor, the cells were serum‐starved and pretreated with vehicle (0.1% DMSO) or JSH (20 µM) for 30 minutes and then control or LPS was added to cells. The cells were fixed at 2 hours post‐LPS treatment, and NF‐кB nuclear translocation was examined via immunofluorescence.

#### 2.5.4 Activation and Inhibition of AP‐1

Cells were serum‐starved and pretreated with vehicle (0.1% DMSO) or AP‐1 inhibitor; T5224 (10 or 20 µM) for 30 minutes and then with TPA (20 or 50 ng/mL) for additional 30 minutes. Phosphorylation of cFOS was assessed via immunoblotting to validate the activation of AP‐1 by TPA and inhibition by T5224.

#### 2.5.5 Progesterone ELISA

hTERT‐HM cells were cultured in serum‐free medium supplemented with 1% ITS and treated with 1 or 10 nM of P4, or its vehicle with control or LPS (1 µg/mL). Medium was then collected 18 hours post‐treatment, and P4 was measured using a P4 ELISA kit according to the manufacturer's protocol (Cayman Chemicals, Cat # 582601).

### 2.6 Protein extraction and immunoblotting

Myometrial tissues were crushed on dry ice, homogenized in lysis buffer [0.08 M Tris/HCl (pH 6.8), 2% SDS, 10% Glycerol] with freshly added protease and phosphatase inhibitor cocktail (Thermo Fisher Scientific Inc) using Tissuelyser II (Qiagen). hTERT‐HM cells were lysed on ice in lysis buffer (same as above), vortexed for 15 seconds twice and incubated on ice for 10 minutes in between. Homogenates from tissues and cell lysates were then sonicated on ice for 10 seconds and centrifuged at 20,000 × *g* for 25 minutes at 4℃, and supernatant was collected and stored at −20℃ till further processing. Protein concentration was determined by BCA (Thermo Fisher Scientific Inc). Immunoblotting was performed as described earlier.^22^ Briefly, equal amount of protein was separated by SDS‐PAGE and transferred to a polyvinylidene difluoride membrane (Trans‐blot Turbo Midi PVDF, Bio‐Rad) using Turbo Trans‐Blot system (Bio‐Rad). After blocking for an hour with 5% milk prepared in TBS‐T, the membranes were incubated with primary antibody at 4℃ for overnight. The membranes were subsequently incubated with horseradish peroxidase (HRP)–conjugated secondary antibody at room temperature for 1 hour. Signals were detected using Luminata HRP substrate (Millipore) or SuperSignal^™^ West Femto Maximum Sensitivity Substrate (Thermo Fisher Scientific), and imaging was performed with ChemiDoc imaging system (Bio‐Rad). Antibodies used for immunoblotting are listed in Table 1. Immunoblotting for ERK2 was used as loading control. Densitometric analysis was performed using Image Lab system (Bio‐Rad).

**TABLE 1:** Sources and dilutions of antibodies used in this study

Antibody	Catalogue #	Dilution	Source
20α‐HSD/AKR1C1	GTX105620	WB ‐ 1:1000 IF ‐ 1:100	GeneTex
SMA	M0851	1:100	DAKO
ERK2	sc‐154	1:2000	Santa Cruz
p‐cFOS	5348	1:500	Cell Signaling
cFOS	sc‐52	1:200	Santa Cruz
Phospho‐IκBα (Ser32/36)	9246	1:500	Cell Signaling
IκBα	4812	1:500	Cell Signaling
NF‐кB	ab16502	1:100	Abcam
Cx43	ab1728	1:1000	Millipore
Goat anti‐rabbit IgG‐HRP	sc‐2004	1:5000‐1:10 000	Santa Cruz
Goat anti‐mouse IgG‐HRP	sc‐2005	1:5000‐1:10 000	Santa Cruz
Horse Anti‐Rabbit IgG Antibody (H + L), Biotinylated	BA‐1100‐1.5	1:200	Vector Labs
Streptavidin, Alexa Fluor™ 594 conjugate	S11227	1:1000	Thermo Fisher
Donkey anti‐Mouse IgG (H + L) Secondary Antibody, Alexa Fluor 488	A21202	1:200	Thermo Fisher
Donkey anti‐Rabbit IgG (H + L) Secondary Antibody, Alexa Fluor 488	A21206	1:300	Thermo Fisher

Abbreviations: WB, Western blotting; IF, immunofluorescence.

### 2.7 Immunofluorescence

Paraffin‐embedded human myometrium tissues were sectioned at 5 μm thickness, and slides were baked at 60℃ overnight. The slides were de‐waxed in xylene; 3 changes for 10, 5 and 5 minutes, respectively, and then rehydrated in the descending grades of ethanol (90%, 80%, 70%, 50% for 2 minutes each). Microwave heat treatment was performed for antigen retrieval using DAKO target antigen retrieval solution (Cat #S2367), followed by treatment with 0.01% Triton X‐100 for 3 minutes. Auto‐fluorescence was quenched using 1% Sudan black solution in 70% ethanol for 1 minute followed by 3 washes in PBS (10 minutes each). Slides were then treated with blocking solution (Dako protein block Cat #X0909) for one hour at room temperature. Incubation with primary antibodies (20α‐HSD and smooth muscle actin (SMA), listed in Table 1) diluted in DAKO Antibody Diluent with Background Reducing Components (Cat # S3022) was done for overnight at 4℃. The next day slides were washed 3 times with PBS (10 minutes each) and secondary antibodies, Alexa Fluor 488 donkey anti‐mouse for SMA and biotinylated horse anti‐rabbit for 20α‐HSD, were applied together for one hour. After another set of PBS washes Streptavidin‐Alexa Fluor^™^ 594 conjugate (for 20α‐HSD) was applied to the slides. DAPI (1:1000; Sigma‐Aldrich, Cat # D9542) was used to stain nuclei and slides were mounted with antifade mounting medium Vectashield, (Vector Labs). Rabbit and mouse IgGs (matched concentration as primary antibodies) were used as negative control.

NF‐кB immunofluorescence staining in cells was performed utilizing a method described earlier.^27^ The hTERT‐HM cells (treated as described above for 2 hours) in chamber slides (Cat # 154534PK, Thermo Fisher Scientific) were fixed with ice‐cold (−20℃) methanol:acetone (1:1) for 3 minutes, washed with PBS thrice, permeabilized with 0.2% Triton X‐100 for 5 minutes, washed with PBS thrice and blocked with 1% bovine serum albumin (BSA) solution prepared in PBS for 1 hour at room temperature. NF‐кB antibody was applied to the cells for overnight at 4℃. Next day, cells were washed three times with PBS (10 minutes on rocking platform) and incubated with Alexa Fluor 488–conjugated donkey anti‐rabbit IgG (H + L) secondary antibody for 30 minutes at room temperature. Cells were washed with PBS and mounted with antifade mounting medium.

Fluorescent microscopy was performed using Leica DM IL LED‐Inverted fluorescence microscope with micropublisher 5.0 RTV Q imaging system or Leica Spinning Disc Confocal Microscope under different magnifications.

### 2.8 RT‐PCR

Total RNA was extracted from cells using RNeasy Mini Isolation Kit (Qiagen, Cat # 74134), according to manufacturer's instructions. 500 ng of total RNA was reverse transcribed using iScript Reverse Transcription Supermix (Bio‐Rad, Mississauga, Cat # 1725035), according to manufacturers’ instructions. Amplification was carried out in a volume of 25 μL containing PCR Mix, (Thermo Fisher Scientific) and 10 pmol each of forward and reverse primers (synthesized by Eurofin Genomics, Toronto, Ontario, Canada). Primer sequences for 20‐αHSD (*AKR1C1)* and glyceraldehyde 3‐phosphate dehydrogenase (*GAPDH;* internal control) are provided in Table 2. Amplification protocol: 95℃ for 30 seconds, 60℃ for 45 seconds and 72℃ for 30 seconds. For all PCRs, an initial step to activate HotStar Taq at 95℃ for 3 minutes and a final extension of 5 minutes at 72℃ were also performed. PCR products were visualized on a 1.5‐2% agarose gel containing SYBR Safe (Thermo Fisher Scientific). The mRNA levels of *GJA1* was determined by quantitative RT‐PCR using SYBR green detection chemistry (Sigma). Expression of *GJA1* was normalized to the geometric mean of three housekeeping genes: tyrosine 3‐monooxygenase/tryptophan 5‐monooxygenase activation protein zeta (*YWHAZ*), *GAPDH*, and TATA box binding protein (*TBP*) using CFX manager 3.1 software (Bio‐Rad Laboratories Ltd.).

**TABLE 2:** Sequences of primers used for RT‐PCR

Gene	Gene Symbol		Primer Sequence (5' to 3')	NCBI Ref. Sequence	Amplicon Size (bp)
20 alpha hydroxysteroid dehydrogenase	*AKR1C1*	Forward	CAGCCAGGCTAGTGACAGAA	NM_001353.6	145^a^
		Reverse	ATTGCCAATTTGGTGGC		
Gap junction protein alpha 1	*GJA1*	Forward	ATGAGCAGTCTGCCTTTCGT	NM_000165.5	249
		Reverse	TCTGCTTCAAGTGCATGTCC		
Glyceraldehyde‐3‐phosphate dehydrogenase	*GAPDH*	Forward	AGATCATCAGCAATGCCTCC	NM_002046.7	92
		Reverse	CATGAGTCCTTCCACGATAC		
Tyrosine 3‐monooxygenase/tryptophan 5‐monooxygenase activation protein zeta	*YWHAZ*	Forward	CCGCCAGGACAAACCAGTAT	NM_003406.4	94
		Reverse	ACTTTTGGTACATTGTGGCTTCAA		
TATA box binding protein	*TBP*	Forward	CCACAGCTCTTCCACTCACA	NM_003194.5	138
		Reverse	CTGCGGTACAATCCCAGAAC		

^a^ Reference^9^.

### 2.9 Identification of putative NF‐кB and AP‐1 consensus binding sites in human AKR1C1 promoter

We used transcription factor binding site (TFBS) search 2.0 which utilizes LASAGNA (Length‐Aware Site Alignment Guided by Nucleotide Association) algorithm (https://biogrid‐lasagna.engr.uconn.edu/lasagna_search/index.php)^28^ to identify the putative NF‐кB and AP‐1 binding sites in the 5′ flanking region of human *AKR1C1* promoter (−886 to +43). Two NF‐кB consensus binding sites were identified at positions −586 to −572 and −324 to −312 and two of AP‐1 sites at −464 to −456 and −417 to −409.

#### 2.9.1 Construction of deletion vectors by site‐directed mutagenesis

A plasmid vector, plightSwitch‐Prom‐AKR1C1 (referred as pWT), containing the human *AKR1C1* (encodes 20α‐HSD) promoter (−860 to +43 bases relative to the transcriptional start site) upstream of the open reading frame encoding firefly luciferase reporter was obtained from Switch Gear Genomics (Product ID: S711489). Deletion vectors for NF‐кB (pan‐кB) and AP‐1 (pΔAP‐1) consensus sites (identified above) were created (Figure 5A) using a site‐directed mutagenesis kit (Agilent Technologies). Primer sequences for introducing desired mutations are provided in Table 3.

**TABLE 3:** Sequences of primers used for mutagenesis of 20α‐HSD promoter

Primer name	Primer sequence (5' to 3')	Deletion
NF‐кB‐distal‐sense	5'‐CAGGACTGCACCTTGATCAGGATCTTACAAGGCTAATAAG‐3'	−586 to −572
NF‐кB‐distal‐antisense	5'‐CTTATTAGCCTTGTAAGATCCTGATCAAGGTGCAGTCCTG‐3'	
NF‐кB‐proximal‐sense	5'‐GCCGCTAGAGGTTTCTGTATTCTTAATTTGTTCTACAAATCTCTTTGA‐3'	−324 to −312
NF‐кB‐proximal‐antisense	5'‐TCAAAGAGATTTGTAGAACAAATTAAGAATACAGAAACCTCTAGCGGC‐3'	
AP‐1‐distal‐sense	5'‐GGGCAATATTTTAAAGAAATGCTCTTCCTTTAAATTGTGTGTGTGAGA‐3'	−464 to −456
AP‐1‐distal‐antisense	5'‐TCTCACACACACAATTTAAAGGAAGAGCATTTCTTTAAAATATTGCCC‐3'	
AP‐1‐proximal‐sense	5'‐AAATTGTGTGTGTGAGAGAGAGAAAAATGAAATTTCAAATGCTGCAAGAT‐3'	−417 to −409
AP‐1‐proximal‐antisense	5'‐ATCTTGCAGCATTTGAAATTTCATTTTTCTCTCTCTCACACACACAATTT‐3'	

#### 2.9.2 Transient transfection and luciferase reporter assay

Transient transfection was performed using Lipofectamine 3000 transfection reagent (Thermo Fisher Scientific), following manufacturer's protocol. HEK293T cells were transiently co‐transfected with pWT and pRSVβgal vector. Cells were allowed to recover 5 hours after transfection by replacement of transfection medium with serum‐free DMEM/F12 supplemented with 1% ITS and treated with P4 or vehicle for 24 hours and then harvested in passive lysis buffer (Promega). Luciferase activity was determined using LightSwitch luciferase assay kit (Active Motif, Switch Gear Genomics) as per manufacturer's instructions. Transfection efficiency was normalized with β‐galactosidase activity. Experiments were performed with at least triplicates for each experimental condition and replicated thrice as independent experiments.

### 2.10 Statistical analysis

The Student *t* test was used to determine the differences between two groups (non‐labouring versus labouring samples or control versus treatment). Differences among several groups were determined by one‐way analysis of variance (ANOVA), followed by Dunnet's multiple comparison test using Prism software (GraphPad Prism). Two‐way ANOVA and Tukey's or Sidak's post‐tests were used to compare different variables. Differences were considered significant only where *P* values were less than .05.

## 3 RESULTS

### 3.1 20α‐HSD is up‐regulated in myometrium during labour

**FIGURE 1:**
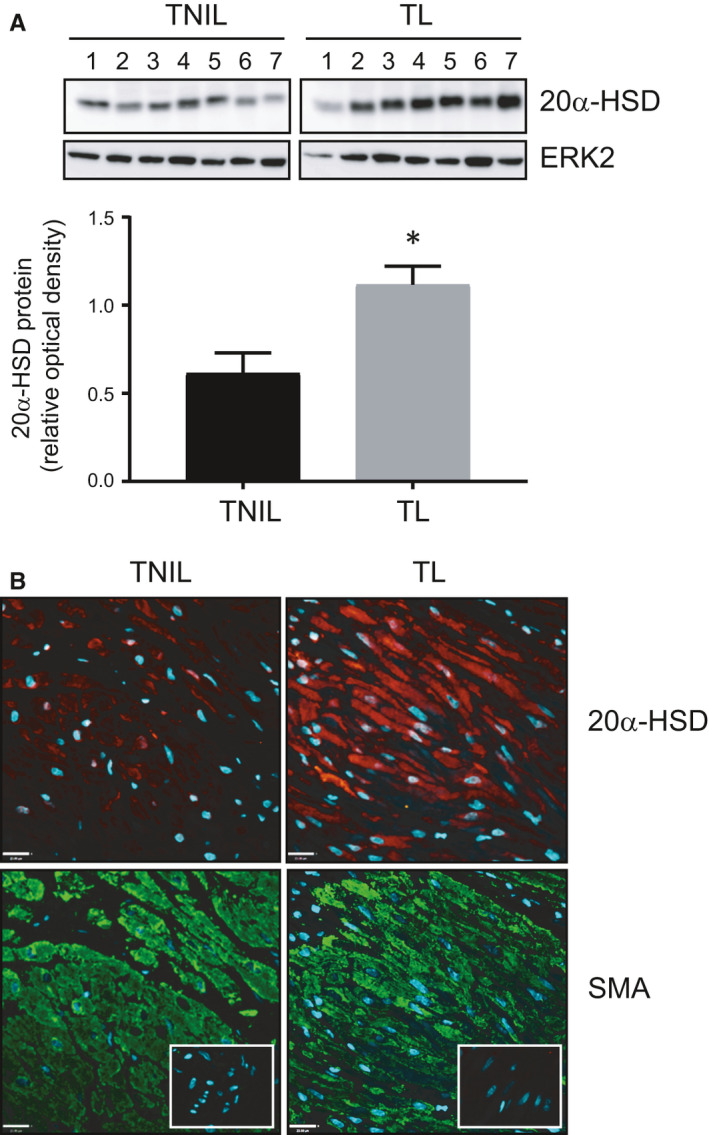
20α‐HSD is increased in human myometrium during labour. A, Representative immunoblots and densitometric analysis of 20α‐HSD protein in human myometrium from term non‐labouring women undergoing elective C‐section (TNIL) and term labouring (TL) women with emergency C‐section. Relative optical density of immunoreactive bands of 20α‐HSD/ERK2 (representing relative 20α‐HSD protein levels) are shown as mean ± SEM, n = 7 each ‘*’ denotes statistical significance at *P* < .05. Unpaired t test was used to determine the differences in protein expression between the TL *vs* TNIL group. B, Representative pictures of immunofluorescence staining of human myometrium tissues from TNIL and TL with anti‐smooth muscle actin (SMA, green) and anti‐20α‐HSD (red) antibodies; nuclei are stained with DAPI. IgG controls are shown as embedded images. Scale bar = 20 μm

**FIGURE 2:**
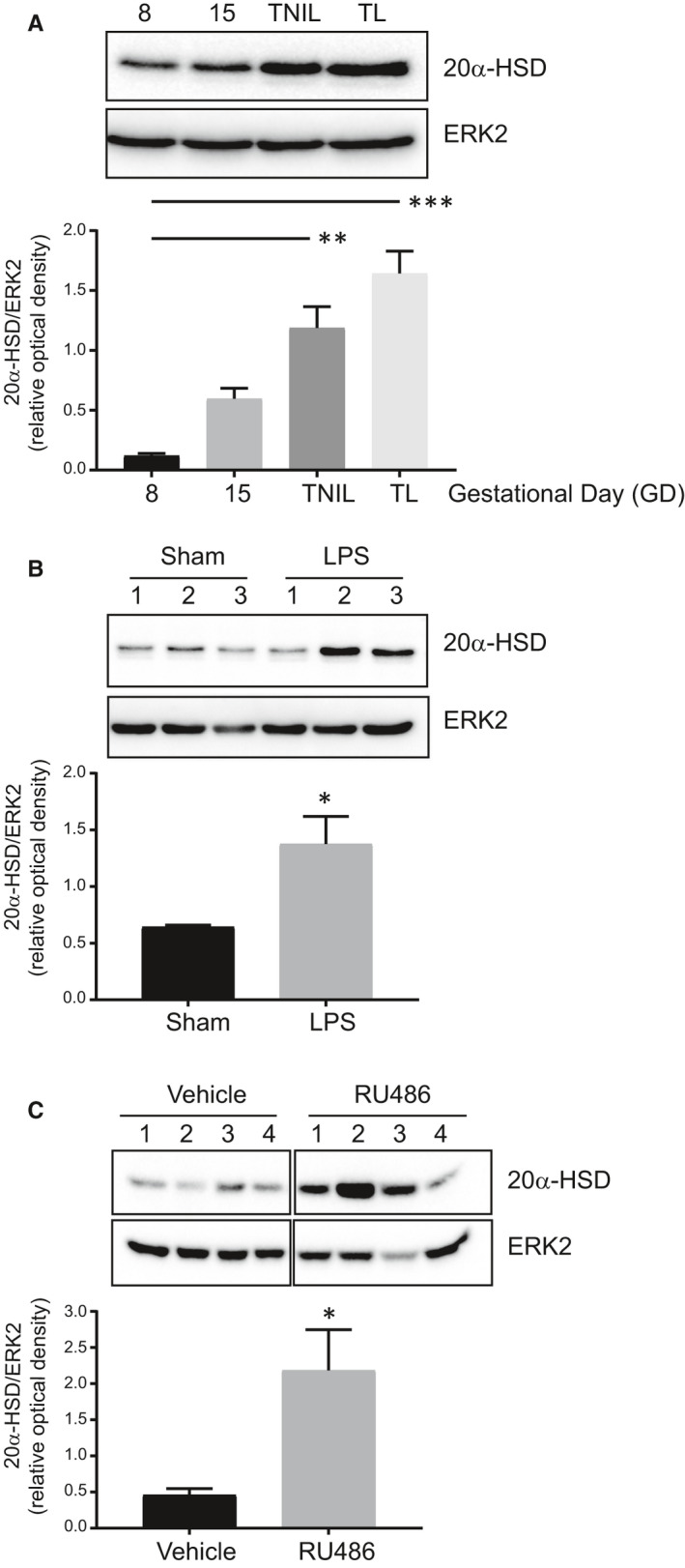
Myometrial 20α‐HSD increases with advancing gestation and during preterm labour in mice. A, Representative Western blots and densitometric analysis of 20α‐HSD protein in mouse myometrium from A) early gestation (gestational day 8, GD8), mid‐gestation (GD15), term not in labour (GD19, TNIL) and term labour (TL). Graph represents mean relative optical density of 20α‐HSD protein with respect to ERK2 ± SEM, n = 4 per GD. ‘**’ denotes statistical significance at *P* < .01 and ‘***’ at *P* < .001 for respective groups vs GD8, determined through ordinary one‐way *ANOVA* followed by Dunnett's multiple comparisons test. B, LPS‐induced preterm labour model where GD15 mice underwent mini‐laparotomy under general anaesthesia with intrauterine infusion of 125 µg LPS in 100 µL of sterile saline (LPS group) or intrauterine infusion of 100 µL sterile saline (Sham group). Graph represents samples from GD16: sham (non‐labouring) and LPS (labouring, n = 3). C, RU486‐induced preterm labour model where GD15 mice received intraperitoneal injection of progesterone antagonist RU486 (150 µg). Graph represents samples from GD16: vehicle (non‐labouring) and RU486 (labouring n = 4). Densitometry data show relative optical density of 20α‐HSD protein with respect to total ERK2, represented as mean ± SEM, ‘*’ denotes statistical significance at *P* < .05. Unpaired t test was used to determine the differences between the treatment vs control group

The onset of labour at term was associated with increased abundance of 20α‐HSD protein in human myometrium as assessed by immunoblotting (*P* = .03, n = 7, Figure 1A) and immunofluorescence (Figure 1B). In mice, 20α‐HSD protein abundance in myometrium assessed by immunoblotting was low during early gestation (GD8) and increased with the advancing gestation (GD15) peaking at GD19 (term not in labour, TNIL) and during TL (Figure 2A). Myometrial tissue collected during PTB induced by LPS or RU486 also had higher levels of 20α‐HSD protein compared with sham/vehicle controls (LPS: *P* = .04, Figure 2B; *RU486*: *P* = .04, Figure 2C).

### 3.2 Pro‐inflammatory stimuli induce human 20α‐HSD expression and activity in vitro

**FIGURE 3:**
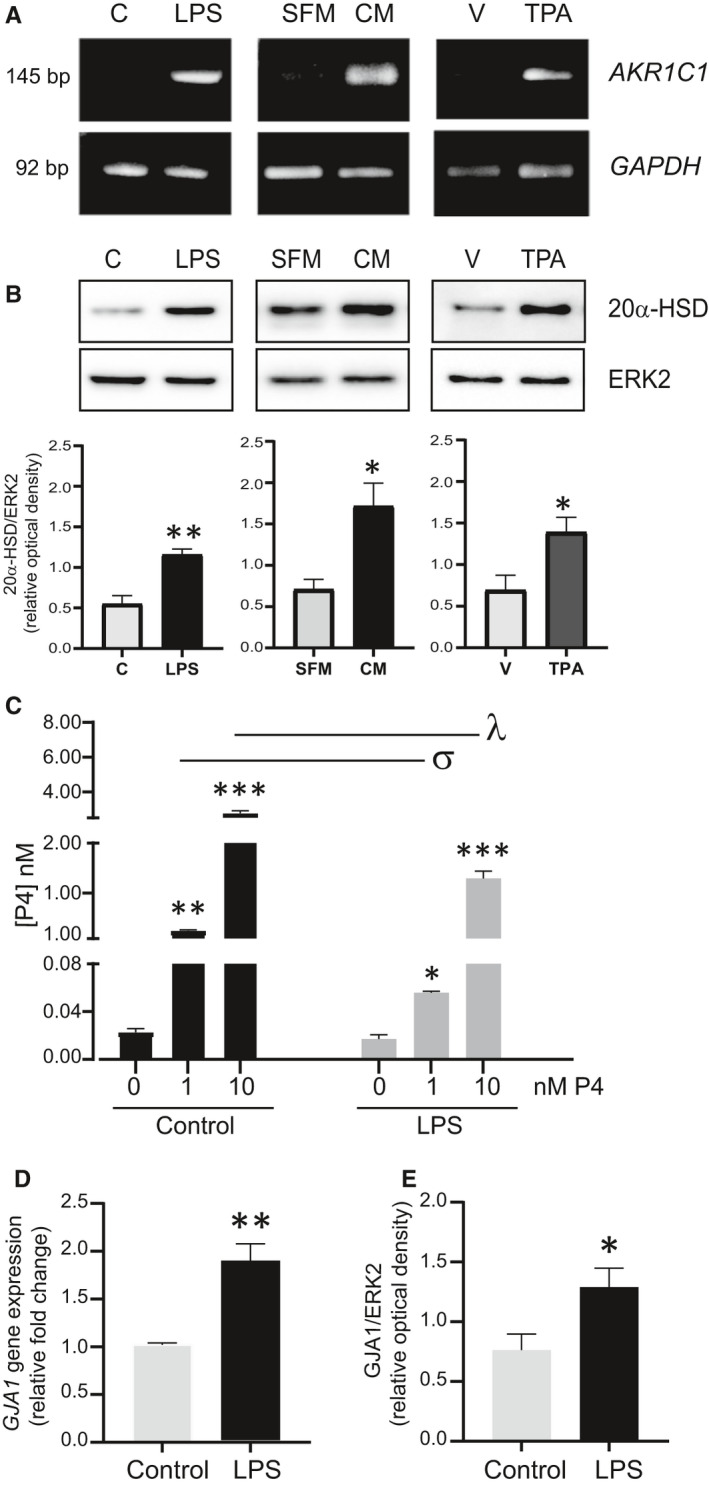
Pro‐inflammatory stimuli induce human 20α‐HSD expression in vitro. hTERT‐HM cells were treated with either LPS (1 µg/mL) or TPA (20 ng/mL) or conditioned medium (CM) from LPS‐treated cells. A, Total RNA was extracted at 4 h post‐treatment, reverse transcribed and subjected to PCR for *AKR1C1* and *GAPDH*. B, Representative Western blots and densitometry analysis of total proteins extracted at 24 h post‐treatment. Data show mean ± SEM (n = 3 for LPS and CM, n = 4 for TPA). Statistical significance was determined by unpaired t test, ‘*’ denotes statistical significance at *P* < .05, ‘**’ at *P* < .01. C, Pro‐inflammatory stimuli induce human 20α‐HSD activity in vitro. Culture medium from hTERT‐HM cells treated with LPS showed depletion of P4 compared to control. hTERT‐HM cells were treated with increasing doses of progesterone (P4; 1 and 10 nM) in the presence or absence of LPS, P4 levels were assessed in the culture medium 18 h post‐treatment. Data show mean ± SEM (n = 3 independent experiments), statistical significance was determined between the treatments (V vs P4) and among the groups (control vs LPS) by two‐way ANOVA followed by Sidak's multiple comparison test comparing control with LPS, ‘*’ and ‘σ’ denote statistical significance at *P* < .05, ‘**’ and ‘λ’ at *P* < .01, ‘***’ at *P* < .001. D, LPS induced the expression of *GJA1* in human myometrium cells. *GJA1* mRNA levels are displayed as fold change, mean ± SEM (n = 3) relative to control. E, GJA1 protein relative optical density with respect to ERK2 is shown as mean ± SEM (n = 4). Statistical significance (D, E) was determined by unpaired t test, ‘*’ denotes statistical significance at *P* < .05, ‘**’ at *P *< .01

LPS and TPA increased the abundance of 20α‐HSD mRNA (Figure 3A) and protein (Figure 3B) in hTERT‐HM cells. To determine the contribution of secreted factors as a potential mechanism of LPS action, the effect of medium conditioned by myometrial cells treated with LPS (LPS‐CM) on *AKR1C1* expression was examined. LPS‐CM increased *AKR1C1* mRNA and 20α‐HSD in hTERT‐HM cells (Figure 3A,B). LPS decreased the recoverable amount of exogenous P4 in hTERT‐HM cell media (Figure 3C). Furthermore, LPS increased *GJA1* mRNA (encodes CX43) (Figure 3D) and GJA1 protein in hTERT‐HM cells (Figure 3E).

### 3.3 Pro‐inflammatory stimuli induce human AKR1C1 promoter activity in vitro, NF‐кB mediates the effect of LPS while AP‐1 transcription factors mediate the effect of TPA on human AKR1C1 promoter activity

**FIGURE 4:**
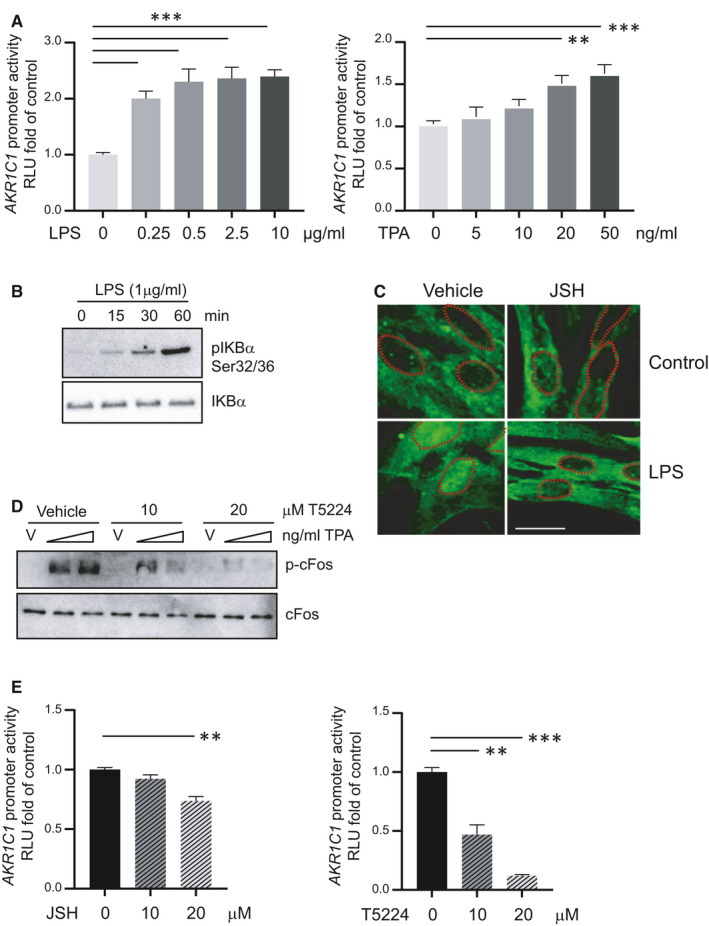
A, Pro‐inflammatory stimuli induce human *AKR1C1* promoter activity in vitro HEK293T cells were transiently co‐transfected with human *AKR1C1* promoter (pWT) and pRSVβgal for overnight and treated with increasing doses of LPS (0, 0.25, 0.5, 2.5 or 10 µg/mL) or TPA (0, 5, 10, 20, 50 ng/mL) for 24 h. B, LPS treatment enhances phosphorylation of NF‐кB inhibitor; IκBα. Representative Western blot showing pIκBα and total IκBα in hTERT‐HM cells treated with 1 µg/mL of LPS for 0, 15, 30 and 60 min. C, Representative images of immunofluorescence (IF) with NF‐кB antibody (green) and nuclei (outlined by dotted orange line), showing nuclear translocation of NF‐кB after LPS treatment and its complete inhibition by treatment with NF‐кB inhibitor (JSH, 20 µM). Scale bar = 20 μm. D, Western validation of activation of AP‐1 transcription factor ‘cFOS’ as determined by its phosphorylation upon TPA treatment, and its inhibition by AP‐1 inhibitor (T5224, 10 µM or 20 µM). Total cFOS is shown as loading control. E, NF‐кB inhibition attenuates LPS‐mediated effects while AP‐1 inhibition abrogates TPA‐mediated effects on *AKR1C1* promoter activity. HEK293T cells were transiently co‐transfected with human *AKR1C1* promoter (pWT) and pRSVβgal for overnight and treated with NF‐кB inhibitor (JSH, 10 or 20 µM) or AP‐1 inhibitor (T5224, 10 or 20 µM), for 30 min alone and then along with LPS (1 µg/mL) or TPA (50 ng/mL) for overnight. The relative luciferase to β‐galactosidase activity is represented by mean ± SEM, as the fold induction from cells treated with vehicle control (group 0). Data are pooled from n = 3 independent experiments performed in triplicate. Statistical significance was determined through one‐way ANOVA followed by Dunnette's multiple comparisons test. ‘**’ denotes statistical significance at *P* < .01 and ‘***’ at *P* < .001. RLU = Relative Luminescence Unit

**FIGURE 5:**
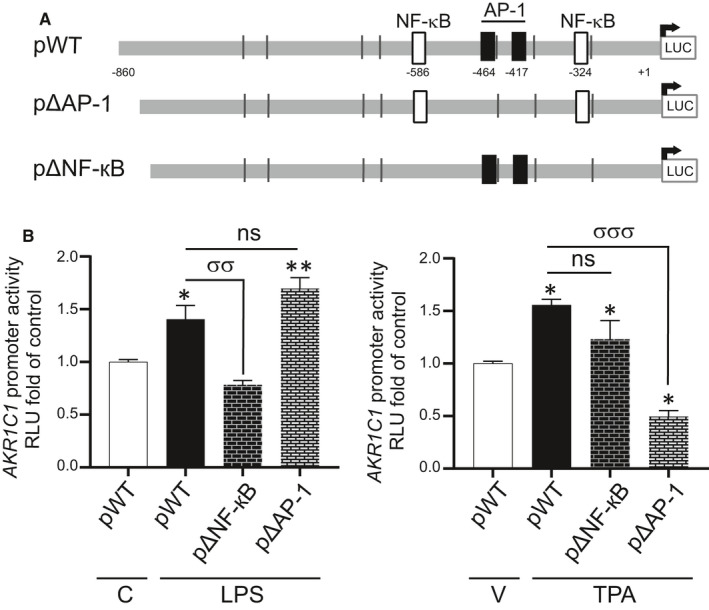
LPS mediates its effects on *AKR1C1* promoter activity via NF‐кB consensus sites, while TPA activates it through AP‐1 binding sites. A, Diagram of human 20α‐HSD/*AKR1C1* promoter‐luciferase reporter construct (pWT) showing number and position of AP‐1 and NF‐кB consensus binding sites in 5′ flanking region of human *AKR1C1 gene* (−860 to +43), and the mutant vectors generated via site‐directed mutagenesis with deletion of AP‐1 sites (pΔAP‐1) or NF‐кB sites (pan‐кB). B, HEK293T cells were transiently co‐transfected with pWT or pan‐кB or pΔAP‐1 and pRSVβgal, treated with control or LPS (1 µg/mL) or vehicle or TPA (50 ng/mL) for 24 h. The relative luciferase to β‐galactosidase activity is represented by mean ± SEM, as fold induction compared to cells treated with vehicle/control. Data are pooled from n = 3 independent experiments performed in quadruplicate. Statistical significance was determined by one‐way ANOVA followed by Tukey's multiple comparisons test. Significance between control (C) and LPS is represented by ‘*’ and between pWT and mutant vectors is shown by ‘σ’. ‘*’ denotes statistical significance at *P* < .05, ‘**’ and ‘σσ’ at *P* < .01 and ‘σσσ’ at *P* < .001. RLU = Relative Luminescence Unit

To assess the transcriptional regulation of human 20α‐HSD by the pro‐inflammatory stimuli, the human 20α‐HSD promoter (−886 to +43) with a luciferase reporter (pWT) was transfected into human HEK293T cells and treated with LPS or TPA. We found that LPS (0.25‐10 µg/mL) and TPA at ≥20 ng/mL were able to induce 20α‐HSD promoter activity above the basal transcription levels (*P* < .05; Figure 4A). We determined that LPS treatment of hTERT‐HM cells induced phosphorylation of NF‐kappa‐B inhibitor alpha (IκBα) protein (Figure 4B) and subsequent nuclear translocation of NF‐кB, which was blocked by the JSH (NF‐кB inhibitor) (Figure 4C). TPA treatment induced phosphorylation and activation of AP‐1 factor cFOS protein, which was blocked by the AP‐1 inhibitor T5224 (Figure 4D). The luciferase activity of LPS‐ and TPA‐induced *AKR1C1* promoter was decreased by NF‐kB and AP‐1 inhibitors, respectively (Figure 4E). LPS‐induced *AKR1C1* promoter‐luciferase activity was significantly reduced by the deletion of the NF‐κB response elements but not by deletion of the AP‐1 response elements (Figure 5B, left). TPA‐induced *AKR1C1* promoter‐luciferase activity was inhibited by deletion of AP‐1 response elements (Figure 5B, right).

## 4 DISCUSSION

Our previous study^2^ and that of Williams et al^1^ suggest that parturition in women is, at least in part, triggered by localized P4 withdrawal. Data in this study support the hypothesis that inflammatory mediators associated with term and preterm labour induce localized P4 withdrawal in myometrial cells by increasing expression of *AKR1C1* and abundance and activity of its protein product 20α‐HSD that metabolizes P4 to an inactive form. Consistent with previous results,^1^, ^2^ we found up‐regulation of myometrial 20α‐HSD during labour in human and mouse myometrium (Figures 1 and 2) and in mouse models of preterm labour (LPS‐model and RU486‐model), which suggest that local P4 metabolism may be critical for myometrial activation and labour onset. Unfortunately, we were unable to obtain myometrial tissues from women in preterm labour to examine 20α‐HSD expression, which could have strengthened the conclusions drawn from the murine models of PTB.

Our in vitro analyses using human myometrial cells (hTERT‐HM) treated with inflammatory stimuli (mimicking infectious or non‐infectious conditions) confirm the positive association of 20α‐HSD levels with inflammation. Notably, the effect caused by LPS treatment itself was similar to the effect of LPS‐CM in the induction of 20α‐HSD levels. LPS‐induced signal transduction leads to secretion of multiple pro‐ and anti‐inflammatory cytokines/chemokines by the treated cells,^29^ which can impart their effects in an autocrine/paracrine manner. Our previous study showed that static mechanical stretch also induces secretion of numerous cytokines and chemokines (eg IL‐6, CXCL8, CXCL1, MIF, VEGF, G‐CSF, IL‐12, bFGF and PDGF‐bb) from the hTERT‐HM cells,^30^ which suggests that uterine stretch may also contribute to 20α‐HSD‐mediated local P4 metabolism.

The consequence of 20α‐HSD up‐regulation is increased metabolism of P4 and reduction in the levels of biologically active P4. In this study, we provide evidence of P4 depletion in the culture medium of human myometrial cells treated with LPS. Since steroids freely move across the cell membrane down their concentration gradient, depletion of P4 in the culture medium of LPS‐treated cells suggests increased metabolism of intracellular P4 within the myometrial cells. These data are consistent with increased 20α‐HSD in response to inflammatory stimuli.

It is known that infection/inflammation regulate the activity of AP‐1 and NF‐кB.^29^ LPS differentially activates AP‐1 and NF‐кB depending upon its serotype.^31^ In this study, we treated hTERT‐HM cells with LPS‐O55:B5 serotype to mimic infection, which resulted in phosphorylation of NF‐кB inhibitor, IκBα, and nuclear translocation/activation of NF‐кB, while treatment with TPA to mimic non‐infectious inflammation caused phospho‐activation of AP‐1 protein cFOS (Figure 4B‐D). It has been reported that LPS acts through Toll‐like receptor 4 to activate downstream signalling in which MyD88/TNFR‐associated factor 6 (TRAF6) phosphorylates IκBα, which results in its dissociation from/and subsequent nuclear translocation/activation of NF‐кB.^32^ TPA has been used as a non‐infectious model of inflammation and is known to induce activity of AP‐1 TFs and *GJA1* expression through the protein kinase C pathway.^22^, ^33^, ^34^, ^35^ Whether it is normal term labour (physiologic inflammation) or infection‐induced PTL, myometrial activation of AP‐1 and/or NF‐кB is observed,^21^, ^36^ and current study suggests that both of these TFs can induce *AKR1C1* promoter activity and therefore might contribute to local P4 withdrawal.

Regulation of 20α‐HSD in reproductive tissues is multi‐faceted. While we determined that NF‐кB and AP‐1 affect the transcription of the *AKR1C1* in the myometrium, studies conducted in other reproductive tissues have shown the involvement of other factors. For example, one of the molecular pathway attributed to the regulation of myometrial expression of *AKR1C1* is the miR200‐STAT5b axis. STAT5b is a transcriptional repressor of *AKR1C1*.^9^ The targeted degradation of STAT5b by miR200 results in de‐repression of *AKR1C1* transcription. However, this negative regulatory axis is under the influence of P4, which up‐regulates TF: ZEB1, to keep the expression of miR200 family in check.^13^

Prolactin is reported to suppress the ovarian expression of *Akr1c18* (rat homologue of *AKR1C1*) during early pregnancy, leading to higher levels of P4.^37^, ^38^ PGF_2α_ mediates activation and up‐regulation of AP‐1 (JUND) and NUR77 TF in the corpus luteum of rats to induce *Akr1c18* and participate in P4 withdrawal and labour onset.^39^ In mouse ovarian cells, *Akr1c18/AKR1C1* transcription is shown to be regulated by Sp‐1 and Sp‐3 TFs^40^ and in human ovaries by NF‐Y/CEBP.^41^

The AP‐1 family of TFs were previously implicated in regulation of 20α‐HSD gene promoter activity in the monkey.^42^ In this study, we determined that the 5′ flanking region of *AKR1C1* (−860 to +43) is responsive to LPS and TPA. Moreover, blocking NF‐кB or AP‐1 activity, using specific inhibitors, attenuated *AKR1C1* promoter activity suggesting that both TFs regulate human *AKR1C1* transcription. The targeted deletion of NF‐кB and AP‐1 consensus sites revealed that NF‐кB mediates infection (LPS)‐induced *AKR1C1* transcription, while the AP‐1 pathway is critical for inflammation (TPA)‐induced *AKR1C1* transcription. Given that both AP‐1 and NF‐кB are activated in myometrium prior to labour,^21^, ^22^, ^36^, ^43^, ^44^ we suggest that these TFs are pivotal in the up‐regulation of 20α‐HSD and consequent localized P4 withdrawal in myometrial cells. We have previously found that the AP‐1 specific dimer composition differentially regulates gene expression and that the activity of nuclear AP‐1 heterodimers (FRA1/JUNB and FRA2/JUND) is associated with human labour.^22^ Elucidation of the specific role of different AP‐1 dimers in the regulation of 20α‐HSD transcription will be important to delineate the molecular regulation of *AKR1C1* expression.

**FIGURE 6:**
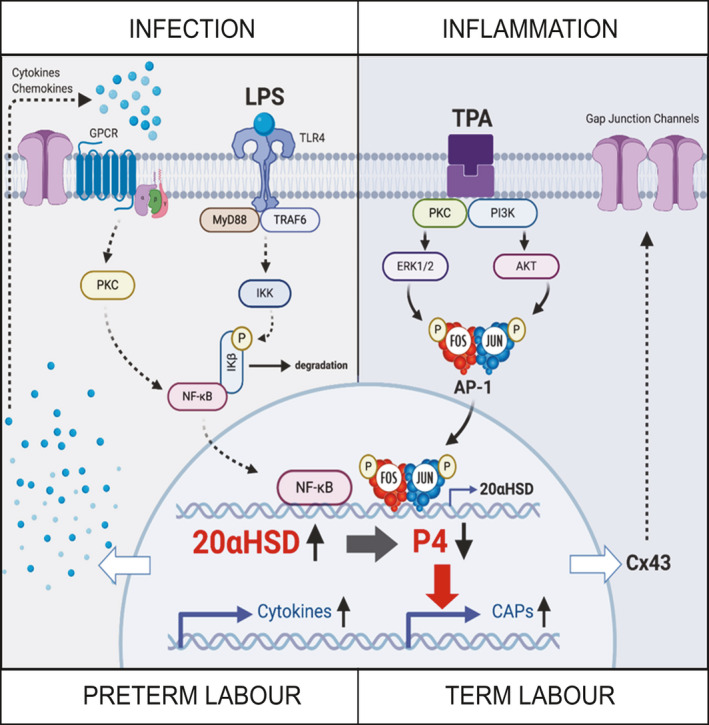
Schematic representation of putative molecular mechanism by which different inflammatory stimuli regulate P4 withdrawal and myometrial activation. 20α‐HSD expression is up‐regulated in myometrium in response to 1) infection (LPS), which activates the NF‐кB transcription factor and mediates its nuclear translocation through phosphorylation and subsequent degradation of IκBα in TLR4/MyD88/TRAF‐dependent pathway, or 2) inflammatory stimuli (TPA), which mediate phosphorylation, activation and translocation of AP‐1 factors via PKC/PI3K pathways. Up‐regulation of 20α‐HSD expression causes decrease of P4 levels and induces transcription of CAPs (such as *GJA1/*Cx43), resulting in enhanced myometrial cell connectivity through the formation of gap junction channels, activation of myometrium and labour onset. The inflammatory signals (LPS and TPA) generate a secondary response via secretion of multiple cytokines and chemokines by myometrial cells to elicit autocrine and paracrine positive feedback loop, thereby amplifying the inflammatory response. Lipopolysaccharide (LPS), 12‐O‐tetradecanoylphorbol‐13‐acetate (TPA), 20alpha hydroxysteroid dehydrogenase (20α‐HSD), nuclear factor‐κB (NF‐кB), activator protein 1 (AP‐1), inhibitor of nuclear factor kappa B (IкBα), Toll‐like receptor 4 (TLR4), myeloid differentiation primary response protein (MyD88), tumour necrosis factor receptor (TNFR)–associated factor 6 (TRAF6), protein kinase C (PKC), phosphoinositide 3‐kinase (PI3K), progesterone (P4), contraction‐associated proteins (CAPs). Note that this figure was created with Biorender.com

Taken together, our findings provide a molecular mechanism linking uterine inflammation and myometrial P4 withdrawal, which precedes labour onset (summarized in Figure 6). We propose that up‐regulation of myometrial *AKR1C1/*20α‐HSD during labour is conserved in term (mice and human) and preterm parturition (mice). We found that inflammatory signals, driven by mimics that are widely accepted as representing infection and inflammation, induce *AKR1C1* expression through the activation of AP‐1 or NF‐кB TFs. The resultant increase in 20α‐HSD may cause local P4 withdrawal that remove inhibition of CAP gene expression, especially *GJA1*, that cause the contraction of labour by increasing myometrial cell contractility and excitability. We suggest that present data on local metabolism of P4 in myometrium explain the conflicting results on the effectiveness of clinical P4 treatment aimed at preventing PTB.^45^, ^46^, ^47^ It may be possible, therefore, to clinically inhibit labour and prevent PTB using anti‐inflammatory therapeutics that inhibit the activation of *AKR1C1* expression and 20α‐HSD activity in myometrial cells, thus preserving the relaxatory and pro‐gestational actions of P4.

## CONFLICT OF INTERESTS

The authors have no conflicts of interest.

## AUTHOR CONTRIBUTIONS

**Lubna Nadeem:** Conceptualization (lead); Data curation (lead); Formal analysis (lead); Funding acquisition (supporting); Investigation (lead); Methodology (lead); Project administration (lead); Supervision (lead); Validation (equal); Visualization (equal); Writing‐original draft (equal). **Rathesh Balendran:** Data curation (supporting); Formal analysis (supporting); Investigation (supporting); Methodology (supporting); Validation (supporting); Visualization (supporting). **Anna Dorogin:** Data curation (supporting); Investigation (supporting); Methodology (supporting); Validation (supporting); Visualization (supporting). **Sam Mesiano:** Conceptualization (supporting); Funding acquisition (supporting); Writing‐review & editing (equal). **Oksana Shynlova:** Conceptualization (lead); Funding acquisition (equal); Investigation (supporting); Methodology (supporting); Project administration (equal); Supervision (equal); Validation (supporting); Visualization (supporting); Writing‐review & editing (lead). **Stephen J Lye:** Conceptualization (lead); Funding acquisition (lead); Project administration (lead); Resources (lead); Supervision (lead); Visualization (equal); Writing‐review & editing (equal).

## Data Availability

The data that support the findings of this study are available from the corresponding author upon reasonable request.
